# 550 The Effect of COVID-19 in Hospital Length of Stay and Patient Population Following Burn Injury

**DOI:** 10.1093/jbcr/irae036.184

**Published:** 2024-04-17

**Authors:** Sara Sheikh-Oleslami, Bettina Papp, Anthony Papp

**Affiliations:** The University of British Columbia, North Vancouver, BC; Douglas College, Burnaby, BC; Vancouver General Hospital, Vancouver, BC; The University of British Columbia, North Vancouver, BC; Douglas College, Burnaby, BC; Vancouver General Hospital, Vancouver, BC; The University of British Columbia, North Vancouver, BC; Douglas College, Burnaby, BC; Vancouver General Hospital, Vancouver, BC

## Abstract

**Introduction:**

Myriad medical services were complicated by the COVID-19 pandemic. Acute burn care is heavily resource dependent, and thus was greatly impacted by lack of specialized personnel, supplies, and other resources which grew scarce with the onset of COVID-19. The primary objective of this study was to examine the relationship between COVID-19 and the length of stay (LOS) in hospital following burn injury as prolonged hospital admission increases opportunity for hospital-related complications and has implications on both the individual and community level. Further, it has been shown that homeless patients are hospitalized longer than housed patients due to lack of respite care and resources post-discharge. As such, the secondary objective of this study was to see how COVID-19 affected the homeless burn population and its possible contribution to LOS.

**Methods:**

Single-centre, retrospective cohort study using data from the Burn Registry and medical chart review with inclusion of all adult burn patients admitted to a quaternary provincial burn unit from April 1, 2016, to March 31, 2023. Patients admitted prior to April 1, 2020, were considered the pre-COVID cohort. Variables of interest included demographic characteristics as well as LOS. Homelessness was defined as lack of fixed address. We compared variables of interest by LOS.

**Results:**

498 patients met inclusion criteria. Of these, 301 fell into the pre-COVID and 197 into the COVID cohorts. The mean age (years) and TBSA (%) (standard deviation, [SD]) were 48 (17) and 12 (13) in the pre-COVID cohort and 47 (15) and 14 (16) in the COVID cohort. Males comprised 75.4% and 75.6% of the pre-COVID and COVID cohorts, respectively. Only 9.3% and 14.2% of patients suffered inhalational injuries in the pre-COVID and COVID cohorts, respectively. Overall, there was no significant differences in LOS between cohorts, with COVID cohort patients staying in hospital for 22 (24) days compared to 20 (29) days in the pre-COVID cohort. However, a 169% increase in homeless patients was seen during COVID, with 16% (49/301) of admitted patients being homeless pre-COVID compared to 27% (53/197) during COVID.

**Conclusions:**

COVID-19 pandemic did not affect the LOS in burn patients, but the results suggest that homeless patients were disproportionately affected by the pandemic.

**Applicability of Research to Practice:**

This is important as prolonged hospital stays have serious implications for the patient and the healthcare systematic as a whole.

